# Review: Genomic insights into the adaptive traits and stress resistance in modern horses

**DOI:** 10.1007/s44154-025-00274-1

**Published:** 2026-01-12

**Authors:** Halima Jafari, Belete Kuraz Abebe, Li Cong, Zulfiqar Ahmed, Wang Zhaofei, Minhao Sun, Gemingguli Muhatai, Lei Chuzhao, Ruihua Dang

**Affiliations:** 1 Key Laboratory of Animal Genetics, Breeding and Reproduction of Shaanxi Province, College of Animal Science and Technology, Northwest A&F University, Yangling, 712100 China; 2 Faculty of Veterinary and Animal Sciences, University of Poonch Rawalakot, Rawalakot, Azad Jammu and Kashmir 12350 Pakistan; 3 College of Animal Science and Technology, Tarim University, Alar, 843300 China

**Keywords:** Adaptive traits, Environmental stressors, Stress resistance, Domestication, Genome selection, Modern horses

## Abstract

The domestication and selective breeding of horses have profoundly influenced the emergence of adaptive traits and stress resistance mechanisms, shaping modern equine populations. This comprehensive review examines the genomic foundations of these traits, emphasizing recent advancements in high-throughput sequencing technologies and bioinformatics. These tools have elucidated the genetic underpinnings of key characteristics such as endurance, speed, metabolic efficiency, and disease resistance. Importantly, the review identifies and connects gene variants associated with thermoregulation, immune function, and cellular repair mechanisms, shedding light on their synergistic roles in enabling horses to adapt to diverse environmental challenges and physiological stressors. By establishing these causal links, this review enhances the coherence between genomic findings and their implications for equine biology. Furthermore, the integration of genomic insights provides a framework for addressing contemporary challenges in horse management and conservation. Issues such as climate change, disease outbreaks, and the preservation of genetic diversity demand innovative strategies grounded in genomics. By bridging the findings on equine adaptation and stress resistance mechanisms with practical applications in breeding and management, this review highlights the potential of genomics to ensure the sustainability and resilience of equine populations in the face of evolving environmental and societal pressures. This expanded perspective underscores the critical role of genomics in both understanding the evolutionary trajectory of horses and guiding future practices in equine health and conservation.

## Introduction

The domestication and selective breeding of horses have played a pivotal role in shaping modern equine species, fostering a wide range of adaptive traits and stress-resistance mechanisms (Orlando 2020; Orlando and Librado 2019). The genus *Equus*, the sole extant genus in the family Equidae, evolved significantly, with *Equus caballus* exhibiting major adaptations in body size, limb, and spinal structure (Cirilli et al. 2022; Solounias et al. 2018). These evolutionary changes, driven by environmental factors and human domestication, have enhanced the horse’s specialized physiological capacity and athletic abilities (Jones 2016). Around 2.5 million years ago, *Equus* migrated from North America to Eurasia, adapting to the diverse environments of the Chinese steppes (de Barros Damgaard et al. 2018; Radovic et al. 2024). Several species, including *Equus stenonis* and *Equus sanmeniensis*, evolved from *Equus simplicidens* (Sun and Deng 2019), while the Debao pony, adapted to environmental stress, exemplifies genetic influences on size, such as the *NELL1* and *TBX3* genes (Asadollahpour et al. 2020). Modern research on Chinese horse breeds has identified genetic markers linked to performance traits, including novel single-nucleotide polymorphisms (SNPs) in the *MSTN* gene (Li et al. 2014).

In 2006, an international collaborative project launched the sequencing of the equine genome, a milestone that provided the first complete genome of a perissodactyl in 2007 and continues to refine our understanding (Kalbfleisch et al. 2018; Wade et al. 2009). Notably, sequencing of the horse *Equus caballus* chromosome 11 (ECA11) centromere highlighted its evolutionary significance (Purgato et al. 2015; Raudsepp et al. 2019; Wade 2013). This research offers valuable insights into mammalian centromere evolution, with implications for breeding practices and genetic diversity (Criscione et al. 2022; Lindsay-McGee et al. 2023; Solounias et al. 2018).

Horses’ vital roles in transportation, agriculture, and warfare have shaped the selection of traits such as endurance, speed, and strength, driving the field of equine genomics (Cosgrove et al. 2020; Librado et al. 2016). Advances in sequencing technologies now allow in-depth exploration of genes related to thermoregulation, metabolic efficiency, and disease resistance (Cappelli et al. 2023; McCue et al. 2015; Todd et al. 2023), shedding light on horses’ remarkable adaptability to environmental and societal pressures. This review synthesizes recent research on the genetic foundations of adaptive traits and stress resistance in horses, highlighting the importance of genomic insights for breeding, conservation, and health optimization. By integrating these findings, we aim to provide a comprehensive understanding of the resilience and performance traits of modern horses in the face of evolving challenges.

## Approaches and methods for screening of selection signals and genomic regions

Currently, there is a growing scientific ability to examine how genetics and the environment intertwine using modern genomic tools, such as reference genomes, genotyping arrays, SNPs, and next-generation sequencing (NGS), allowing for a deeper understanding of complex trait interactions (Howard et al. 2017). The Equine Genetic Diversity Consortium (EGDC) was established to comprehensively assess nuclear diversity and relationships within and among horse populations on a genome-wide scale. Numerous studies have been conducted on genetic diversity and selection pressures in livestock populations (Babayi et al. 2021; Bazvand et al. 2024; Delgado et al. 2019; Dementieva et al. 2023; Khalt-Abadi and Moradi 2018; Nogueira et al. 2022). The first comprehensive study of the equine genetic diversity among large breeds was presented by Petersen et al. (2013), which helped to establish a foundation for equine genetic research. Whole genome sequencing (WGS) stands out as a preferred method for scrutinizing traits in highly selected breeds due to its versatility and global applicability (Durward-Akhurst et al. 2024; Metzger et al. 2014). WGS allows for the comprehensive analysis of genetic variation across entire genomes, particularly effective for identifying SNPs, copy number variations (CNVs), and structural variants that contribute to adaptive traits. It is globally applicable and versatile, making it the preferred method for examining highly selected breeds (Durward-Akhurst et al. 2024; Metzger et al. 2014). SNP arrays are widely used for genotyping large populations. The Equine SNP50 BeadChip and SNP70 genotyping arrays enable genome-wide association studies (GWAS) and CNV detection. For example, the Equine SNP50 Bead Chip in Thoroughbred identified markers related to race distance aptitude (Kim et al. 2018). In contrast, SNP70 arrays uncovered copy number variation regions (CNVRs) in Mongolian horses (MG), Debao ponies (DB), and Yili horses (YL), identifying trait-associated genes such as *CIDEB*, *FGF11*, *PPARG*, *HIVEP1*, *SPEM1*, and *GALR* (Kader et al. 2016a, b). Another CNVR-based study in the Pura Raza Espanola (PRE) breed revealed genes related to scent transduction, olfactory receptor activity, and immune function, enhancing the understanding of equine genetics and phenotypic variation (Laseca et al. 2022). The Copy Number Ratio (CNR) detects the gains or losses in genomic regions relative to a reference, aiding in the evaluation of genomic integrity and variation (Choudhury et al. 2023; Ghosh et al. 2014, 2016).

GWAS have been instrumental in identifying variants associated with particular traits or risk alleles. SNP-based genotyping arrays have revealed numerous loci involved in heritable conditions, such as lavender foal syndrome, an autosomal recessive neurological disorder characterised by coat color dilution and early mortality (Alkalamawy et al. 2018; Ayad et al. 2021). Another example is Laryngeal neuropathy, a respiratory disease that affects the athletic performance of horses (Boyko et al. 2014; Brooks et al. 2018).

Selection signals and allele frequencies are determined through different methods, such as fixation index (F_ST_) and cross-population composite likelihood ratio (XP-CLR), which capture genomic regions under selection between breeds. F_ST_ measures allele frequency differences between populations, with typical thresholds ranging from 0.2 to 0.5, where higher values indicate stronger differentiation. Software such as Arlequin and PLINK are often used for F_ST_ calculations (Asadollahpour et al. 2019; Asadollahpour and Kharrati-Koopaee 2021; Lee et al. 2018). For example, F_ST_ analysis revealed genomic regions under selection in Debao ponies, linked to their unique small body size and endurance traits (Asadollahpour et al. 2019). Similarly, XP-CLR detects selective sweeps by comparing haplotype structures across population, and has been used to identify selection signals associated with sport performance traits in Thoroughbreds (THB) and Arabian horses (AR) (Lee et al. 2018).

Meanwhile cross-population extended haplotype homozygosity (XP-EHH) and integrated haplotype score (iHS) identify genes associated with traits within a specific population by analyzing haplotype structure (Ardestani et al. 2020; Santos et al. 2021). iHS values, normalized to a standard scale, are typically considered significant when exceeding ± 2. For instance, iHS uncovered immune-related genes under selection in Warmblood horses, revealing adaptation to specific environmental conditions (Santos et al. 2021).

The nucleotide diversity (π) ratio quantifies polymorphisms within a species and plays a critical role in assessing genomic diversity (Evrigh et al. 2018; Köseman et al. 2019). For inbreeding estimation, runs of homozygosity (ROH) islands are utilized to identify genes associated with desired traits, aiding in the detection of genomic regions under selection and predicting quantitative trait loci (QTL) (Bazvand et al. 2024; Bhardwaj et al. 2023; Chen et al. 2023; Gmel et al. 2024; Mon et al. 2024; Szmatoła et al. 2022). In Swedish Warmblood horses, ROH analysis revealed islands associated with sports performance, present in over 85% of genotyped (Ablondi et al. 2019).

In the domain of epigenomics techniques, such as histone modification, DNA methylation, regulatory RNAs, chromatin immunoprecipitation sequencing (ChIP-Seq), bisulfite sequencing, and RNA sequencing (RNA-Seq) are essential for studying gene regulation. Studies have highlighted the importance of histone modifications (Cappelli et al. 2023; Horvath et al. 2022), DNA methylation patterns (Corbin et al. 2020), and the role of microRNA in gene regulation affecting muscle development and immune responses in horses (Stefaniuk and Ropka-Molik 2016). ChIP-Seq enables analysis of protein-DNA interactions (Kingsley et al. 2019), while RNA-seq quantifies gene expression levels and identifies differential gene expression in response to environmental stresses (Foury et al. 2023; Li et al. 2024; Roberts et al. 2023). Each technique offers unique advantages, and together, they contribute to a deeper understanding of selection signals and genomic adaptation in horses. These integrative approaches are summarized in Table 1.

**Table 1:** Summary of genes associated with local adaptation of domestic horses

Hores Breed	Parameter	Platform	Associated Genes	Ref
Yakut	Cold	WGS	*THRAP3*	(Librado et al. 2015)
Tibetan	High altitude	WGS	*EPAS1*	(Liu et al. 2019)
Jinjiang	Heat-tolerance	CNV	*HSPA1A*	(Wang et al. 2022)
Brazilian	Heat-tolerance	GWAS	*GFOD1, KLF9*	(de Faria et al. 2022)
MG & THB	Racing	WGS	*MYLK2, NTM*	(Han et al. 2022)
HV & DP	Racing	GWAS	*ACTA1*	(Asadollahpour et al. 2019, 2021)
Italian trotter	Racing	SNPs Genotyping	*COX412, GCK*	(Dall’Olio et al. 2021)
THB, Anglo-Arabian	Racing	GWAS, SNPs-based	*MSTN*	(Hill et al. 2019; Pira et al. 2021)
Chinese domestic	Racing	PCR–RFLP	*MSTN*	(Li et al. 2014)
Quarter horse	Racing	GWAS	*AKNA*, *ARMC2*, *ZFP37*, *HNRNPU*	(Pereira et al. 2019)
Arabian horses	Racing	R-PCR and PCR–RFLP	*SH3RF1, SH3RF2, ACTN3*	(Ropka-Molik et al. 2018, 2019a, b)
Mangalarga Marchador	Racing	GWAS	*PPP4R2*, *PDZRN3*, *IFNAR1*, *LOC100071438*	(Littiere et al. 2020)
Baicha Iron Hoof	Hoof health	Genome-wide SNPs	*CSPG4, PEAK1, EXPH5, WWP2, HAS3*	(Han et al. 2023)
Puerto Rican	Gait	GWAS	ECA 23 *DMRT3*	(Wolfsberger et al. 2022)
MG, IT & FT	Gait	GWAS, CSS	*DMRT3*	(Dall’Olio et al. 2021; Han et al. 2023; Ricard and Duluard 2021)
Shetland pony	Body size	WGS, CNVRs	*DIAPH3*	(Metzger et al. 2018)
Noriker	Body size	ROH based	*ZFAT*, *LASP1*, *LCORL/NCAPG*	(Grilz‐Seger et al. 2019)
DP	Body size	GWAS, SNPs based	*TBX3*	(Kader et al. 2016a, b; Liu et al. 2022)
Korean native Jeju	Body size	RNA-seq based	*ACTN2, LCORL, MSTN, LASP1, ZFAT, PDK4, HMGA2*	(Srikanth et al. 2019)
IRH	Hight stature	GWAS	*HMGA2*, *LLPH*	(Mousavi et al. 2023)
JTH	Body weight	GWAS	*MSTN*, *LCORL, TRIB2*, *ZFAT*	(Tozaki et al. 2017)
IT & IRH	Wither height	SNPs Genotyping	*LCORL*	(Dall’Olio et al. 2021; Mostafavi et al. 2019)
Thoroughbred	Muscle growth	WGS, ROH	*ADAMTS15*, *QKI*	(Chen et al. 2023)
AR, MG & THB	Muscle growth	WGS (CSS)	*HDAC9, KTN1, MYLK2, SLC16A1*, *SYNDIG1*	(Han et al. 2022)
Mongolian	Muscle growth	RNA seq based	*MSTN*	(Budsuren et al. 2022)
ABC	Curly hair	GWAS, RNA-seq	*KRT25*, *SP6*	(Thomer et al. 2018)
Yakut	Hair growth	WGS, F_ST_, CNV	*BARX2*	(Librado et al. 2015)
American Paint	Splashed & spotting	WGS, Exon seq	*MITF, PAX3*	(Henkel et al. 2019; Magdesian et al. 2020; Patterson et al. 2022)
Sarcidano & Kushum	Bay, black, chestnut	mtDNA Haplotypes	*MC1R*, *ASIP*	(Cosso et al. 2022; Nguyen et al. 2020)
German Riding Pony	White-spotted	PCR, Sanger seq	*KIT*	(Hug et al. 2019)
Purebred Spanish	Pearl coat	SNP, genotyping	*MATP/SLC45A2*	(Holl et al. 2019; Marín Navas et al. 2022)
Polish Konik	Blue dun	Genotyping based	*TBX3*	(Cieslak et al. 2021)
Noriker	Leopard spotted	ROH based	*MC1R, PATN1*	(Gril‐Seger et al. 2019)

## Response to stress and adaptivity to cold and high-altitude environments

Horses, as warm-blooded animals, have mechanisms to maintain a stable body temperature despite external climate changes (Cao et al. 2023; Holcomb 2017; Proops et al. 2019). Their remarkable ability to adapt to cold weather involves various physiological and behavioral stress responses that enable them to thrive in cold and frozen environments (Mejdell et al. 2020; Williams et al. 2015; Yudin et al. 2017). In extremely cold regions, such as the Siberian Far East, genome studies of Yakut horses have identified cis-regulatory mutations (using F_ST_ analyses), particularly in genes linked to rapid phenotypic adaptations (Librado et al. 2015). These include the development of hairy winter coats, changes in body size, and alterations in metabolic pathways, all of which are significantly to their survival at temperatures below −70℃ (Plemyashov et al. 2022).

Research has shown that genes like *ATP1A2*, *CYP11B2*, *HSPG2*, *ACADSB*, and *THRAP3* are associated with temperature regulation and cold adaptation in domestic horses (Librado et al. 2015). *ATP1A2*, which encodes a subunit of the sodium–potassium pump, plays a crucial role in maintaining ionic homeostasis and cellular energy balance under thermal stress. Its activity supports the regulation of body temperature during prolonged exposure to cold environments by optimizing cellular metabolism (Manigandan and Yun 2023). Similarly, *CYP11B2*, involved in aldosterone biosynthesis, regulates electrolyte balance and blood pressure, which are critical for maintaining thermoregulation in cold conditions (Takeda et al. 2023). Horses with genetic variants in these genes exhibit increased physiological resilience, enabling them to conserve body heat effectively.

In addition, *HSPG2* and *ACADSB* contribute to adaptive thermogenesis and cellular protection under extreme temperatures. *HSPG2* encodes perlecan, a heparan sulfate proteoglycan that plays a role in extracellular matrix stability and tissue protection, which may enhance cold resilience by preserving tissue integrity during frost exposure (Ma et al. 2020). *ACADSB*, involved in the beta-oxidation of branched-chain fatty acids, supports energy production under conditions of low ambient temperature, ensuring sustained metabolic activity (Vermillion et al. 2015). Furthermore, *THRAP3*, a transcriptional coactivator, has been linked to stress-responsive gene expression, which may modulate physiological responses to cold environments (Katano-Toki et al. 2013).

These genetic adaptations collectively enable domestic horses to thrive in frigid climates, as demonstrated by their ability to accumulate snow on their backs without melting due to minimized heat dissipation (Osthaus et al. 2018). This reflects not only their advanced physiological mechanisms but also the potential for identifying genomic markers that inform breeding strategies for improved thermoregulation and climate resilience. This adaptation enhances insulation provided by subcutaneous fat, skin, and a thicker hair coat, which can increase by more than 200% in native ponies to withstand extremely low temperatures (Osthaus et al. 2018; Plemyashov et al. 2022). Changes in body composition, particularly in the distribution of adipose tissue, also play a crucial role in their ability to endure low temperatures (Shawaf et al. 2018). Wild equids like Przewalski horses are well adapted to cold climates, with adaptations controlled by noncoding and protein-coding genes and gene duplication (Librado et al. 2015). These horses inhabit regions such as Central Asia and the Tibetan Plateau, characterized by vast plains and rolling mountains (Kaczensky et al. 2020; Ransom and Kaczensky 2016).

Horses’ adaptation to high altitudes involves a multifaceted genomic and physiological framework that supports survival in environments characterized by low oxygen availability, extreme temperatures, and limited resources. Specific genetic mechanisms underpin these adaptations, enabling domestic horses, particularly Tibetan horses, to thrive at altitudes exceeding 5,000 m on the Qinghai-Tibetan Plateau (Cao et al. 2023). The identification of critical genes, such as *EPAS1* and *HBE1*, emphasizes their role in regulating oxygen homeostasis and hemoglobin function (Liu et al. 2019). These genes are not only crucial for Tibetan horses but also show striking parallels to adaptive mechanisms in other high-altitude species, including Sherpa humans (Horscroft et al. 2017), Tibetan dogs (Wang et al. 2014), and yaks (Bai et al. 2024). Such comparisons reveal convergent evolution as a recurring theme in high-altitude adaptation, highlighting how similar selective pressures shape physiological resilience across diverse taxa.

In addition, adaptive evolution in mitochondrial genes (e.g., *NADH6*, *ND6*, *ATP8*, and *CYTB*) enhances metabolic efficiency under hypoxic conditions (Yang et al. 2018). These findings align with studies on Andean camelids and Andean horses, where mitochondrial adaptations also play pivotal roles in supporting aerobic metabolism in low-oxygen environments (Gutiérrez et al. 2023). Cross-species analyses of genetic pathways, such as those involving the hypoxia-inducible factor (HIF) family, further underscore the universality of certain adaptive strategies while highlighting species-specific genetic signatures shaped by localized evolutionary pressures (P. Zhao et al. 2024).To enhance the persuasiveness of our arguments, we have incorporated additional references comparing horses’ genetic adaptations to those observed in other mammals. For example, the cytochrome *P450 3A* gene family, strongly associated with high-altitude resilience in Andean horses, demonstrates the interplay between stress response, toxin metabolism, and hypoxia tolerance (Hendrickson 2013). These cross-species parallels reinforce the importance of metabolic pathways and oxygen regulation as central pillars of high-altitude survival.

The relationships among physiological, genomic, and behavioral adaptive mechanisms in high-altitude horses highlight a dynamic interplay between different strategies for coping with environmental stressors. For instance, thermoregulation is a critical physiological adaptation that involves both genetic and behavioral mechanisms. Genomic analyses have identified genes that promote efficient energy production and fat metabolism, crucial for maintaining body temperature in cold climates (Wonghanchao et al. 2024). These genetic adaptations are complemented by behavioral strategies, such as huddling in groups to conserve heat and minimize energy expenditure, demonstrating how physiological and behavioral mechanisms synergize to optimize survival.

Similarly, the interaction between oxygen regulation and cellular repair mechanisms illustrates a multifaceted response to hypoxia. Hypoxia-inducible factors (e.g., those regulated by *EPAS1*) not only enhance oxygen transport but also activate pathways involved in cellular protection and repair, such as antioxidant responses and mitochondrial biogenesis. These processes are critical for mitigating oxidative stress caused by low oxygen levels, ensuring cellular integrity and metabolic stability.

To further elucidate these relationships, we have expanded our discussion to integrate examples of how physiological adaptations, such as increased red blood cell production and enhanced capillary density, are supported by genetic mechanisms like those observed in *EPAS1* and *HBE1* (Liu et al. 2019). Concurrently, behavioral adaptations, such as grazing patterns optimized for nutrient-rich vegetation, complement these physiological changes by ensuring adequate energy intake in resource-scarce high-altitude regions.Stress resistance in high-altitude horses encompasses responses to both hypoxic and thermal stress, with genetic and physiological adaptations working in tandem. For example, cold stress induces metabolic changes that are supported by genetic adaptations in mitochondrial function. The upregulation of genes such as *ND6* and *ATP6* enhances ATP production, ensuring sufficient energy supply for thermogenesis (Yang et al. 2018). Behavioral adaptations, such as seeking shelter or altering activity patterns during extreme weather, further reduce energy expenditure and mitigate the effects of cold stress.

Moreover, genomic indicators such as ROH and CNVs reveal how genetic diversity contributes to stress resilience. These genetic markers are associated with enhanced regulation of hypoxia-inducible factors and improved cellular repair mechanisms, underscoring their role in maintaining physiological stability under environmental stress (Liu et al. 2019). Comparative studies of other high-altitude species, such as Andean camelids and Tibetan dogs, highlight similar patterns of genetic variation, further illustrating the convergent evolution of stress resistance mechanisms.

## Physiological and genetic adaptations to heat stress

Horses demonstrate a variety of physiological and genetic adaptations to manage heat stress, which are critical for maintaining homeostasis and productivity in hot environments. These adaptations include evaporative cooling through increased sweating, improved respiratory function, and cardiovascular adjustments, supported by a robust genomic framework that enables resilience under heat stress conditions.

Sweating is the primary mechanism of heat dissipation in horses, accounting for up to 65% of total body cooling (Kang et al. 2023). Horses possess an exceptional capacity for sweating, facilitated by their highly efficient apocrine sweat glands. Sweating increases evaporative heat loss, particularly in breeds like Arabian and Marwari horses, which have been exposed to prolonged heat stress over generations. Elevated respiratory rates also play a critical role, allowing horses to dissipate excess heat through respiratory water loss. This mechanism is particularly important during intense exercise, where cardiovascular adjustments increase blood flow to the skin, promoting heat dissipation (Ebisuda et al. 2024).

Behavioral adaptations complement these physiological mechanisms. For example, horses instinctively seek shade or modify their activity patterns during periods of peak solar radiation (Holcomb et al. 2014). Such behavioral strategies reduce core body temperature and mitigate the adverse effects of heat stress.

Genetic factors underpin the physiological adaptations observed in heat-resistant horse breeds. Heat shock proteins (HSPs), particularly *HSPA1A*, play a vital role in protein stabilization and protection under thermal stress. *HSPA1A* prevents protein denaturation and aggregation by acting as a molecular chaperone, ensuring cellular integrity during prolonged heat exposure (Wang et al. 2022). Functional studies have shown that *HSPA1A* expression increases significantly in heat-stressed horses, correlating with improved thermoregulation and stress resilience.

Genes involved in the immune response, such as *NFKBIA* and *SOCS4*, also contribute to heat resistance by modulating inflammatory reactions triggered by elevated temperatures. *NFKBIA* inhibits NF-κB signaling, reducing excessive inflammation that can exacerbate heat stress-induced tissue damage (Benjamin et al. 2019). Similarly, *SOCS4* regulates cytokine signaling pathways, minimizing inflammatory responses and maintaining physiological stability under heat stress conditions.

Inflammatory markers like *IL6*, known for their role in thermoregulation, further exemplify the interplay between genetic and physiological mechanisms. *IL6* expression increases during heat stress, promoting vasodilation and enhancing heat dissipation through improved blood flow (Wang et al. 2022). Together, these genes form an intricate network that supports cellular function, immune balance, and thermoregulatory efficiency in horses.

Chinese Jinjiang and Indian Marwari horses are exemplary models of heat resistance, displaying breed-specific adaptations to high-temperature environments. Genomic studies using Equine BeadChip technology have identified critical genes associated with heat stress tolerance in these breeds. For instance, Jinjiang horses exhibit higher expression levels of *HSPA1A* and *NFKBIA*, correlating with their ability to withstand high ambient temperatures (Wang et al. 2022). Marwari horses, native to the arid Marwar region, demonstrate similar upregulation of *SOCS4* and *IL6*, which enhance their inflammatory response and thermoregulatory capacity under extreme heat (Gupta et al. 2014).

Functional validation studies comparing Jinjiang and Marwari horses under controlled heat stress conditions reveal that both breeds exhibit significant upregulation of heat shock protein genes and inflammatory markers, supporting their genetic predisposition to heat resistance (Liu et al. 2023; Pal et al. 2020; Wang et al. 2022). However, breed-specific differences in gene expression levels highlight the influence of localized environmental pressures. For example, Marwari horses show enhanced sweating efficiency, attributed to their leaner body composition and superior heat dissipation mechanisms, while Jinjiang horses demonstrate greater reliance on immune modulation and protein stabilization (Bhardwaj et al. 2023). The relationship between genetic and physiological adaptations underscores the complexity of heat resistance in horses (Sneddon 2023). Heat shock proteins not only stabilize cellular structures but also interact with inflammatory markers to optimize thermoregulation. For example, the chaperone activity of *HSPA1A* reduces oxidative damage during heat stress, enabling sustained cardiovascular and respiratory function (Abd-El-Aziz et al. 2022). Similarly, the anti-inflammatory effects of *NFKBIA* and *SOCS4* prevent overheating by minimizing tissue damage and promoting efficient heat dissipation through vasodilation (Brownlow and Mizzi 2022; Lee et al. 2018)**.**

**Fig. 1:**
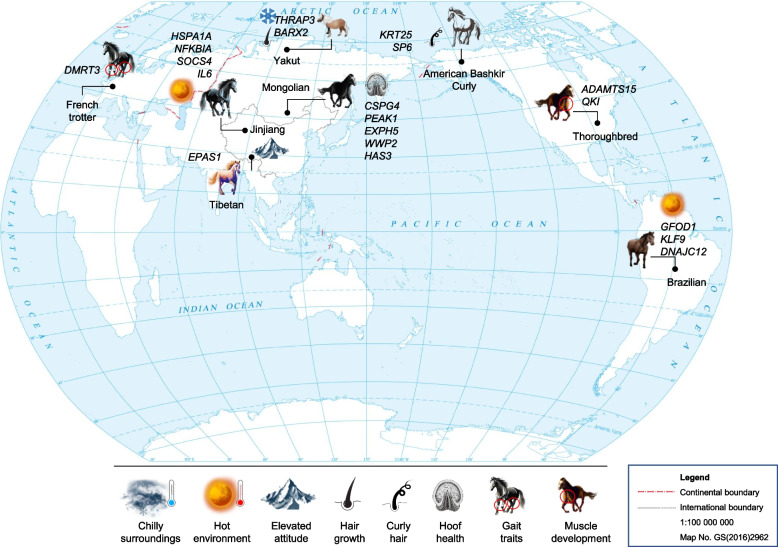
Global distribution of horse breeds and key genes underlying trait-specific adaptations. This figure illustrates the worldwide distribution of representative horse breeds, highlighting genomic adaptations associated with distinct environmental and performance traits. Colored icons denote specific adaptive traits: American Bashkir Curly for curly hair; Brazilian and Jinjiang breeds for heat tolerance; Yakut horse for cold resistance in Siberian Yakutia; Tibetan horse for high-altitude adaptation; Thoroughbred for enhanced muscle development; Mongolian horse for hoof resilience; and French Trotter for gait performance. The figure underscores the interplay between geographical location, environmental selection pressures, and genetic mechanisms that shape trait-specific adaptations across diverse equine populations

Breed-specific adaptations further illustrate the interplay between genetics and physiology. Arabian horses, with their efficient sweating mechanisms and leaner body composition, exemplify how genetic predispositions (e.g., *HSPA1A*) complement physiological traits to enhance heat resistance (Sneddon 2023). In contrast, breeds adapted to temperate climates, such as Thoroughbreds, rely more on moderate sweating and metabolic adjustments, reflecting their limited exposure to extreme heat stress (Putnová and Štohl 2019). As shown in Fig. 1, environmental pressures shape specific genomic patterns that contribute to these adaptations.

## Morphological features and responses to environmental stress

### Coat Color Variations

Coat color in horses is a critical adaptive trait influenced by natural selection factors, including camouflage, UV protection, pathogen resistance, and reproductive success (Marín Navas et al. 2022). Basic coat colors such as black, bay, and chestnut are determined by the epistatic interaction of *MC1R* and *ASIP* genes. The dominant *MC1R* triggers eumelanin production, resulting in darker coats, while recessive alleles produce pheomelanin, resulting in chestnut coats (Neves et al. 2017). *ASIP*, as an antagonist to *MC1R*, controls the distribution of pigment, contributing to bay coloration (Daverio et al. 2016).

Environmental adaptation via coat color is evident in certain breeds and species. For example, black coats in *Lama glama*, resulting from recessive *ASIP* alleles, have been linked to increased melanin synthesis in response to UV radiation, providing better protection against solar damage (Bacon et al. 2023; Zhao et al. 2018). In horses, lighter coat colors may reflect selective pressures favoring camouflage in arid regions or enhanced heat reflectivity (Oyebanjo et al. 2022). The *STX17* gene is associated with the grey phenotype, characterized by depigmentation and increased melanoma susceptibility, illustrating trade-offs between pigmentation and health risks (Rosengren et al. 2008). Coat color can also influence reproductive success through sexual selection. Mutations in *KIT* and *PAX3*, linked to white and spotted phenotypes, may affect mating preferences by increasing visual distinctiveness (Hug et al. 2019; Magdesian et al. 2020; Patterson Rosa et al. 2022). These traits are influenced by environmental factors and breeding practices, underscoring the importance of coat color in adaptation and survival.

The integration of body size, coat thickness, and coat color demonstrates how morphological traits collectively support environmental adaptations. For example, larger body size in cold climates is often accompanied by thicker coats, providing additional insulation, while smaller body size in hot climates correlates with lighter coats that reflect solar radiation and reduce heat absorption (Neves et al. 2017). The interplay between coat thickness and color is particularly evident in breeds adapted to extreme environments. Icelandic horses, for instance, exhibit thick coats and dark colors, optimizing heat retention in cold climates, whereas Arabian horses have thinner, lighter-colored coats suited to heat dissipation in arid regions (Choudhury et al. 2023).

Genetic mechanisms regulating these traits often overlap, with pleiotropic genes like *LCORL* and *HMGA2* influencing both growth and metabolic efficiency. Similarly, pigmentation genes such as *ASIP* and *MC1R* may indirectly affect thermoregulation by altering heat absorption and UV protection. These interactions highlight the interconnectedness of morphological traits and their collective role in environmental stress resistance.

### Body size traits

The evolution of horse body size reflects adaptive radiation influenced by environmental transitions from forests to grasslands and human domestication (McHorse et al. 2019; Shoemaker and Clauset 2014). Body size impacts thermoregulation, metabolic efficiency, and locomotion, which are critical for environmental stress responses. Large body size is advantageous in cold climates due to reduced surface area-to-volume ratios, aiding in heat conservation, while smaller body sizes are better suited for hot climates as they facilitate efficient heat dissipation (Bazvand et al. 2024; Sjaastad et al. 2016).

Genomic studies provide insights into the molecular mechanisms underlying body size adaptations. Positive selection on loci such as *LCORL*/*NCAPG* on ECA3 and *LASP1* on ECA11 has been identified through population differentiation indices (F_**ST**_), linking these genes to growth and skeletal traits (Gurgul et al. 2019; Metzger et al. 2013). The *LCORL* gene influences skeletal growth, height, and carcass composition, with polymorphisms showing significant associations with body size across breeds (Mostafavi et al. 2019; Tozaki et al. 2017). Notably, *HMGA2*, a gene with pleiotropic effects, influences height and metabolic traits in horses, humans, and dogs (Frischknecht et al. 2015; Liu et al. 2020; Norton et al. 2019). For instance, a nonsynonymous mutation in *HMGA2* reduces height in Shetland ponies, demonstrating the gene’s role in small breed adaptations (Frischknecht et al. 2015).

The relationships between body size traits and environmental stress responses are complex. Smaller horses, such as Jeju or Caspian breeds, exhibit adaptations linked to reduced resource availability and climate variations, with selection signatures on genes affecting muscle composition, thermoregulation, and energy metabolism (Srikanth et al. 2019; Mousavi et al. 2023). In addition, genes such as *ADAM17*, *SOX11*, and *KLF11* regulate locomotor traits and reflect adaptations to specific terrains and climates (Krebs et al. 2024). CNV research has further linked body size variations to environmental pressures, highlighting the role of resource quality and climatic challenges in shaping morphological adaptations (Srikanth et al. 2019).

## Genes associated with the racing performance of domestic horses

Temperament traits in horses are shaped by genetic and environmental factors, with significant implications for training, performance, and adaptability. The domestication process has selected for behavioral flexibility, favoring traits like reduced reactivity and increased trainability, which are critical for human interaction and various equestrian disciplines (Wilkins et al. 2014; Briefer et al. 2019). The hypothalamic–pituitary–adrenal (HPA) axis plays a central role in regulating the stress response, linking temperament to physiological resilience (Powell et al. 2023). Recent studies employing genomic approaches, such as Illumina HiSeq2500 and GWAS, have identified key genes influencing temperament. For example, *MAOA* and *AR* variants are associated with behavioral traits, including excitability and calmness, aiding in the identification of horses suitable for racing or recreational purposes (Song et al. 2017; Velie et al. 2018). In addition, *NTM*, one of the top genes selected during domestication, influences cognitive functions such as learning and memory, playing a vital role in equine temperament and performance (McGivney et al. 2019, 2020).

In Mongolian horses, a nonsynonymous mutation (*G217 A*) has been linked to temperament traits reminiscent of early domestication, reflecting their heightened awareness and adaptability to environmental stressors (Ren et al. 2017). These traits, essential for survival in harsh environments, also underscore the competitive edge of Mongolian horses in endurance-based activities. Mongolian horses exemplify the connection between temperament traits, environmental adaptability, and genetic underpinnings, providing a bridge to understanding how these traits influence racehorse breeding. The connection between temperament and racing performance is most evident in traits like excitability, focus, and stress resilience, which directly influence trainability and athletic outcomes. Warm-blooded horses, known for their spirited nature and heightened reactivity, are better suited for high-speed activities, whereas cold-blooded horses exhibit calmer temperaments and are used for slower, heavy work (de Faria et al. 2022; Nolte et al. 2019).

Genomic studies have identified several genes associated with racing performance, highlighting the role of temperament traits in endurance, speed, and stress management. For instance, *ACTA1* is associated with racing ability in Hanoverian horses (HV), particularly influencing skeletal muscle contraction during high-speed activities (Asadollahpour et al. 2019). Arabian horses, known for their endurance and balanced temperament, exhibit genetic variations such as *SORCS3* and *SLC39A12*, linked to cardiac rhythm and stress response during long-distance riding (Ricard et al. 2017; 2020; Ropka-Molik et al. 2019b).

The *MSTN* gene, critical in Thoroughbreds, determines race distance suitability through allele variations, with the homozygous “C” allele enhancing short-distance performance and the homozygous “T” allele favoring long-distance racing (Bryan et al. 2019; Kis et al. 2023). This gene not only influences muscle fiber composition but also impacts metabolic efficiency, highlighting its role in stress adaptation during races (Binns et al. 2010; Rivero and Hill 2016). Positioning Mongolian horses before racehorse genetics enhances the logical flow by emphasizing the broader applicability of temperament traits across equine species. Mongolian horses, adapted to extreme environments, demonstrate the genetic and behavioral traits necessary for endurance and stress resilience. These traits serve as a foundation for understanding how temperament and genetic factors influence specialized breeds like Thoroughbreds and Arabians. For example, both Mongolian and racehorses rely on genes such as *ACTN3*, *PPARα*, and *FOXO3*, which regulate muscle performance and oxidative stress management, aiding in endurance and recovery (Ropka-Molik et al. 2017). Similarly, the *DMRT3* mutant allele, known as the “gait-keeper,” enhances neuromuscular coordination, a critical trait for both competitive racing and adaptation in challenging terrains (Jäderkvist et al. 2014, 2015).

**Fig. 2:**
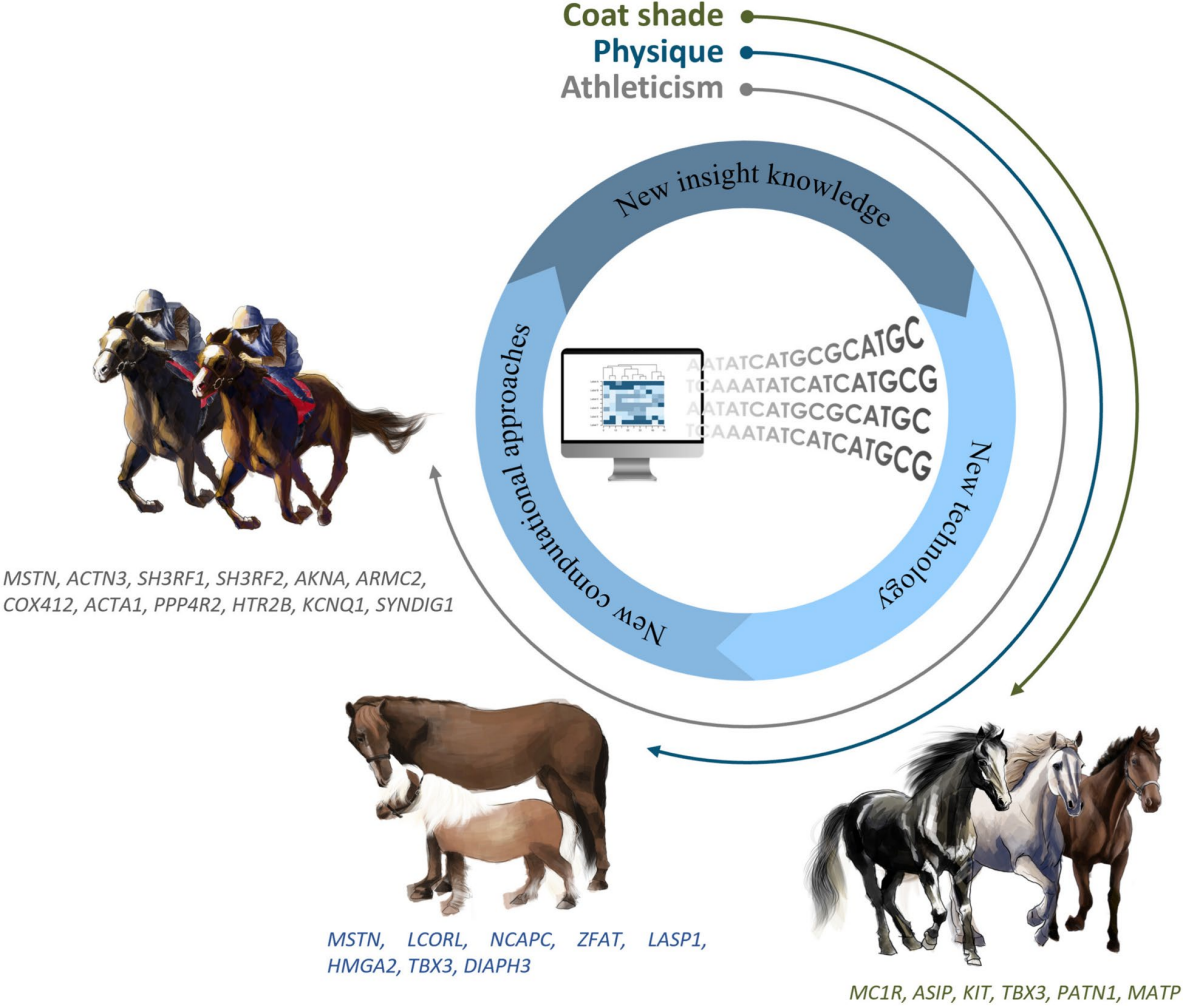
Integration of modern genomic tools for identifying genetic markers associated with key equine traits. This figure provides an overview of the application of advanced genomic technologies in unraveling the genetic architecture underlying important equine traits, such as coat color, body size, and athletic performance. Specific marker genes associated with each trait are highlighted. The circular framework illustrates recent advancements in equine genomics, encompassing high-throughput sequencing platforms, genotyping arrays, and emerging computational methodologies, including GWAS, CNV analysis, and RNA sequencing. Collectively, these approaches have enhanced our understanding of how genetic variants influence complex phenotypic traits in horses

The integration of temperament and performance traits into selective breeding programs has significant implications for equine sports and work disciplines. The identification of genes influencing excitability, focus, and stress resilience provides tools for optimizing training and enhancing performance. For example, transcriptomic studies in Arabian horses reveal that genes such as *ACTN3*, *TGFBR1*, and *TNNC1* are key markers for selecting individuals with superior stress adaptation during races (Stefaniuk and Ropka-Molik 2016). Moreover, advancements in genomic technologies, such as long-read sequencing, have uncovered new candidate genes (e.g., *FIG4*, *HNRNPU*) linked to racing performance, opening avenues for precision breeding to enhance traits like muscle strength, metabolic efficiency, and stress resistance (Dall’Olio et al. 2021; Pereira et al. 2019; Maniego et al. 2023). These findings demonstrate the interconnectedness of temperament, physical traits, and genetic adaptations across equine species, as illustrated in Fig. 2. Advances in sequencing technology and computational biology have made it possible to identify genes responsible for phenotypic traits in horses. This process combines genomic data with novel analytical approaches to reveal genetic variants linked to coat color, body size, and athletic performance, which highlight how modern tools can provide deeper insights into complex traits, more precise and informed breeding strategies.

## Important genes associated with temperament

The domestication process involves selective breeding for temperament traits, resulting in noticeable differences between wild and domestic animals. A crucial component in this process is the hypothalamic–pituitary–adrenal (HPA) axis, which regulates the stress response (Wilkins et al. 2014). Palaeogenomics studies have shown that domestication selects for behavioral flexibility in horses, indicating a focus on neurobiological genes (Briefer et al. 2019). The Illumina Hiseq2500 platform and GWAS have identified *MAOA* (c.1164 + 41 T > C) and *AR* (c.1047 + 27G > T) as significant variants for assessing horse temperament and identifying exceptional race or riding horses (Song et al. 2017; Velie et al. 2018). Notably, *NTM* is among the top 10 genes selected during horse domestication, affecting learning and memory (McGivney et al. 2019, 2020), and playing a significant role in the Thoroughbred racing phenotype (Han et al. 2022). In modern times, horses exhibit signs of selection in chromosome regions containing genes linked to cognitive development and behavioral traits (Wickens and Brooks 2020). Behavior, a crucial quantitative trait influencing performance, work, recreation, and human interaction, has been associated with various genes in different horse breeds (Powell et al. 2023; Wickens and Brooks 2020).

A nonsynonymous mutation (G217 A) in Mongolian horses has been associated with temperament traits reminiscent of early domestication, while the same variant is linked to competitive traits in this breed (Ren et al. 2017). This suggests that horses with heightened awareness and stress adaptability should continue to be favored, underscoring the ongoing importance of these temperament traits in horse breeding practices (Holtby et al. 2023).

## Adaptation to diseases and infections

Horses have evolved a variety of genetic and immunological adaptations to combat viral, bacterial, and parasitic diseases. This section categorizes these adaptations by disease type to provide a clearer understanding of how genetic variations, such as those in *MHC* and *Toll-like receptors ****(****TLRs****)***, contribute to disease resistance.

Viral Diseases: Horses are susceptible to several viral pathogens, and genetic adaptations in immune-related genes play a significant role in combating these infections. Equine herpesviruses (EHV-1 and EHV-4) are significant viral pathogens affecting horses, causing respiratory disease, abortion, and neurologic syndromes. Variations in *MHC* class I and II genes are critical for antigen presentation and immune response to EHV (Plasil et al. 2023). Horses with broader *MHC* diversity tend to have more robust antiviral responses, improving their ability to mount effective immune defenses (Viļuma et al. 2017). Equine Viral Arteritis (EVA) is caused by equine arteritis virus (EAV) and leads to respiratory illness and abortion in infected horses. Genetic variations influence susceptibility, particularly in stallions that serve as carriers. Variations in the *CXCL16* gene, a chemokine involved in antiviral responses, have been implicated in differing susceptibility to EVA (Balasuriya et al. 2018). The innate immune response to West Nile Virus (WNV) relies heavily on *TLRs*, particularly *TLR3*, which recognizes viral RNA and initiates antiviral signaling pathways. Horses with specific *TLR3* polymorphisms exhibit enhanced resistance to WNV, demonstrating the importance of innate immunity in controlling viral infections (Klumplerova et al. 2020). Bacterial Diseases: Genetic adaptations also play a key role in the immune response to bacterial pathogens, particularly through mechanisms involving *TLRs*, *MHC* genes, and mobile genetic elements. Strangles is a highly contagious bacterial disease caused by *Streptococcus equi*, resulting in abscess formation in the lymph nodes. The dynamic genome of *S. equi* indicates ongoing adaptation to equine hosts (Harris et al. 2015). Horses with specific *MHC* class II haplotypes have been shown to have a more effective immune response against *S. equi*, reducing the severity of infections (Klumplerova et al. 2020). Equine Methicillin-Resistant *Staphylococcus aureus* (MRSA) infections are a growing concern, particularly in hospital environments. Genetic elements such as the Staphylococcal Pathogenicity Island (SaPIbov5) and β-hemolysin-converting phages enhance the adaptability and virulence of MRSA in equine hosts (Albert et al. 2019). These mobile genetic elements also promote resistance to antimicrobial treatments, complicating infection control (Jung et al. 2017; Walther et al. 2009, 2018). The immune response to bacterial Lipopolysaccharide (LPS), a component of gram-negative bacteria, involves *TLR4* signaling. While no significant variations in *TLR4* or its co-receptor *MD2* have been linked to differential LPS responses, other *TLR* family members exhibit balancing selection, preserving immune diversity (Migdał et al. 2020; Mukhopadhyay et al. 2023).

Parasitic Diseases: Parasitic infections are a major health concern in horses, particularly in regions with high parasite burdens. Genetic adaptations in the immune system have been identified as critical for managing these infections. Strongylosis (Large Strongyles), caused by parasitic nematodes, leads to significant gastrointestinal damage in horses. The immune response to strongyle infections involves the activation of *TLR5*, which recognizes bacterial flagellin and triggers inflammatory signaling pathways. Evolutionary adaptations in *TLR5* and downstream signaling molecules enhance the immune response to strongyles, reducing parasite load (Pezzanite et al. 2024; Piel & Hart 2024). Equine Protozoal Myeloencephalitis (EPM), caused by the protozoan *Sarcocystis neurona***,** leads to neurological deficits in infected horses. Genetic studies suggest that horses with specific *MHC* class II haplotypes are better able to present protozoan antigens to T cells, resulting in a more effective immune response (Plasil et al. 2023).The gut microbiota plays a pivotal role in modulating the immune response to infections. Species such as *Ligilactobacillus* in the equine gut possess cellulose-degrading enzymes, aiding in digestion and influencing resistance to gastrointestinal pathogens (Chaucheyras-Durand et al. 2022). Genetic variations in gut microbial communities have been associated with differences in disease susceptibility, highlighting the importance of microbiome-immune system interactions (Garber et al. 2020).

## Summary of the genetic makeup of horses in the modern era

Modern horses exhibit significant genetic diversity, but selective breeding has led to varying levels of intra-breed genetic homogeneity and inter-breed differentiation. Studies reveal a trend of prevalent inter-breeding among certain breeds, such as Quarter Horses, Paints, and Tennessee Walking Horses, alongside diminished intra-breed diversity (Solé et al. 2019). At the population level, genomic studies suggest that most modern horses descend from three ancestral lineages: *Equus przewalskii*, *Equus tarpan*, and *Equus robustus* (Der Sarkissian et al. 2015; Do et al. 2014). Among these, only Przewalski’s horse persists today, and understanding its genetic relationship with domestic horses is crucial for reconstructing domestication history (Gaunitz et al. 2018). The advent of NGS and the horse reference genome (Kalbfleisch et al. 2018; Wade et al. 2009) has enabled detailed analyses of genetic diversity. For example, ROH analyses across diverse breeds have identified candidate genes associated with neurotransmission, muscle development, cardiac function, insulin secretion, and spermatogenesis (Chen et al. 2023). These findings are critical for developing conservation strategies and optimizing breeding programs.

Selection for performance traits is evident in racing breeds, where the *MSTN* gene on ECA18 plays a pivotal role in muscle fiber composition and racing ability (Hill et al. 2019; Rooney et al. 2017). Variants of the *DMRT3* gene on ECA23, known as the “gait-keeper,” are strongly associated with alternative gaits in breeds like Icelandic horses and Tennessee Walking Horses (de Oliveira et al. 2020). Body size is another trait shaped by selection pressures. For example, draft breeds like Soviet Heavyweights exhibit selection signatures on ECA11, associated with genes influencing body size and strength (Gurgul et al. 2019). In Italian Bardigiano horses, ROH islands overlap with QTLs linked to conformation traits and disease resistance, reflecting the influence of both natural and artificial selection (Ablondi et al. 2020). Coat color traits illustrate the relationship between selection and environmental adaptation. In Konik horses, selection for dun coat color (a phenotype associated with camouflage) has been linked to the *TBX3* locus on ECA8 (Cieslak et al. 2021). Similarly, CNV analysis in PRE horses identified genes related to pigmentation, behavior, and olfactory receptor activity, underscoring the multifaceted impact of genetic variation on phenotypic traits (Laseca et al. 2022).

Studies of European breeds reveal extensive genomic diversity alongside specific adaptations. For example, the PRE horse, bred for dressage and saddle work since the fifteenth century, exhibits unique genomic signatures associated with temperament, learning ability, and physical conformation (Poyato‐Bonilla et al. 2022). A survey of CNVs across European breeds identified regions overlapping with QTLs related to fertility, behavior, and pigmentation, highlighting the diverse functional impact of genetic variation (Solé et al. 2019). Brazilian breeds, such as Mangalarga Marchador, demonstrate high genetic diversity but also show elevated inbreeding levels due to isolated breeding practices (Nogueira et al. 2022). In Criollo Argentino horses, a breed with origins in natural mating over 500 years ago, genomic analysis revealed 165 autosomal CNVs and 87 CNV regions, including novel ones, providing insights into adaptation and breed history (Corbi-Botto et al. 2019).

**Fig. 3:**
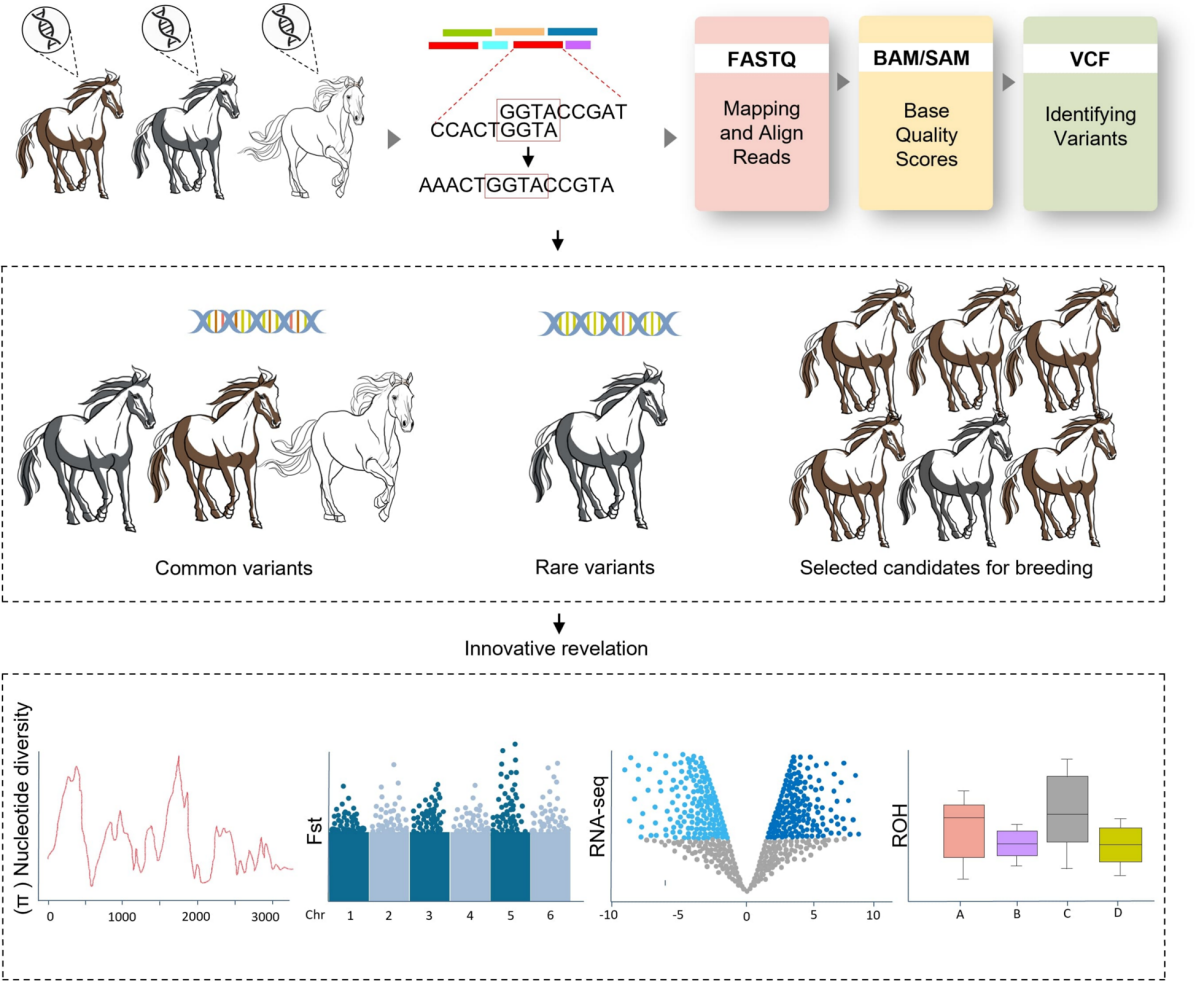
Conceptual pipeline for genomic selection in horse breeds. This figure illustrates the integrated workflow for selecting horse breeds based on genomic technologies. The process begins with the collection of high-quality genomic and phenotypic data, followed by computational analyses including variant calling and data processing (e.g., FASTQ, BAM/SAM, and VCF formats). Population-level analyses are then conducted to identify genetic markers associated with key traits such as performance, environmental adaptability, and stress resilience. These markers inform the development of predictive models for estimating genomic estimated breeding values (GEBVs). The resulting models are applied across broader populations to identify and select superior individuals for breeding, facilitating the targeted enhancement of desirable traits in future generations

A conceptual overview of high-throughput sequencing technologies and genomic tools is presented in Fig. 3 to identify trait-associated genetic variants in horses. Starting with DNA samples, deep sequencing enables genome-wide variant discovery, which can then be analyzed across populations to detect patterns linked to physical and behavioral traits. This process has been central to understanding how selection has shaped the modern horse genome.

## Conclusion and future perspectives

This comprehensive review has highlighted significant advancements in understanding the genetic basis of adaptive traits and stress resistance in modern horses. High-throughput sequencing technologies and bioinformatics have provided transformative insights into the genetic architecture of desirable equine characteristics, such as endurance, speed, and disease resistance. By uncovering the roles of gene variants involved in thermoregulation, immune function, and cellular repair, researchers have shed light on the evolutionary processes that shaped equine populations and have informed contemporary breeding practices aimed at optimizing horse health, performance, and genetic diversity. Looking ahead, the integration of cutting-edge technologies and novel approaches presents exciting opportunities for advancing equine genomics.

The future of equine genomic research lies in leveraging integrative omics approaches that combine genomics with transcriptomics, proteomics, and metabolomics. These multi-omics frameworks enable a deeper understanding of how genetic variations are translated into phenotypic traits across different biological levels. For instance, transcriptomics can reveal gene expression patterns associated with adaptive traits, while metabolomics provides insights into biochemical pathways underlying energy metabolism and stress resistance. Integrating these data streams will facilitate the discovery of novel molecular mechanisms and their interactions in equine adaptation. Emerging technologies like single-cell transcriptomics and spatial transcriptomics offer unprecedented resolution for studying gene expression at the cellular level. Single-cell transcriptomics enables the identification of cell-specific expression profiles, elucidating how individual cell types contribute to stress resistance and adaptation. Spatial transcriptomics adds an additional layer by mapping gene expression to specific tissues or regions, providing insights into the spatial dynamics of adaptive traits, such as muscle development, thermoregulation, and immune responses. These technologies will be instrumental in revealing the cellular and tissue-specific molecular mechanisms driving resilience in modern horses.

Deep learning techniques have shown significant promise in improving the accuracy and efficiency of SNP detection and analysis in horses. These algorithms can process large-scale genomic data, identify complex patterns, and predict associations between SNPs and adaptive traits with greater sensitivity and precision. By employing neural networks and machine learning models, researchers can better analyze genomic datasets to uncover hidden correlations and prioritize candidate variants for functional studies. This approach is particularly valuable in identifying polygenic traits, such as endurance and disease resistance, which involve complex genetic interactions. Mendelian randomization has emerged as a powerful tool for establishing causal relationships between genetic variations and phenotypic traits. By leveraging genetic variants as instrumental variables, this approach minimizes confounding factors, providing robust evidence for the causal effects of specific genes on adaptive traits. For example, Mendelian randomization can be applied to study the influence of candidate genes on stress resistance, immune function, and metabolic efficiency, offering a clearer understanding of their biological roles. Incorporating this method into equine genomic research will enhance the reliability of findings and inform targeted breeding strategies. Functional validation of candidate genes identified through GWAS and genomic analyses remains a critical step in equine genomics. Tools such as CRISPR-Cas9 and base editing enable precise manipulation of genetic sequences, allowing researchers to assess the effects of specific variants on phenotypic traits. These gene-editing technologies hold the potential to confirm causal relationships between genetic variations and adaptive traits, paving the way for future applications in improving equine health and performance. Epigenetics provides a deeper understanding of how environmental stimuli and stressors regulate gene expression. Investigating DNA methylation, histone modifications, and non-coding RNAs will shed light on the mechanisms of phenotypic plasticity and heritable changes that do not involve alterations in the DNA sequence. These studies are particularly relevant for understanding how modern horses adapt to changing environments and management practices, contributing to the development of epigenetic biomarkers for breeding and health monitoring.

Expanding genomic studies to include underrepresented horse breeds and populations worldwide is essential for capturing the full spectrum of equine genetic diversity. Such efforts will provide insights into the evolutionary history, adaptation processes, and unique traits of diverse breeds, informing conservation strategies for endangered populations and maintaining the genetic diversity necessary for sustainable breeding programs. The implementation of genomic selection in equine breeding programs offers a powerful approach to accelerate the improvement of adaptive traits and stress resistance. By utilizing genomic data to predict the genetic merit of individuals, breeders can make more informed decisions, optimizing traits such as endurance, fertility, and disease resilience. Precision breeding strategies based on genomic selection will enhance the sustainability and competitiveness of equine industries. Understanding the interactions between genetic factors and environmental or management conditions is critical for optimizing horse care and performance. Studies that explore how environmental variables influence gene expression, stress responses, and health outcomes will enable the development of tailored management strategies. For example, insights into the genetic basis of heat tolerance can inform optimal training schedules and housing conditions in hot climates, promoting horse welfare and maximizing athletic potential. The continued integration of advanced technologies, such as high-sensitivity omics, deep learning, and gene editing, with population-level studies and functional validation will revolutionize our understanding of equine adaptation and evolution. By uncovering the molecular mechanisms underlying resilience and adaptive traits, these approaches will drive the development of innovative breeding and management practices, ensuring the sustainability and resilience of modern horse populations in the face of environmental and industry challenges.

## Data Availability

Not applicable.

## Abbreviations

SNP: Single nucleotide polymorphism
F_ST_: Fixation index
ROH: Runs of homozygosity
CNVs: Copy number variations
LD: Lower linkage disequilibrium
GWAS: Genome-wide association study
XP-CLR: Cross-population composite likelihood ratio
GGP: GeneSeek Genomic Profiler
WGS: Whole Genome Sequencing
CSS: Composite selection signals
ITHs: Italian Trotter Horses
QTL: Quantitative Trait Loci
XP-EHH: Extended haplotype homozygosity
iHs: Integrated haplotype score
π: Pi ratio nucleotide diversity
CNR: Copy Number Ratio
MG: Mongolian horses
DB: Debao pony
YL: Yili horse
PRE: Pura-Raza Espanola
AR: Arabian
THB: Thoroughbreds
IT: Italian
FT: French trotter
HV: Hanoverians
IRH: Iranian horses
ABC: American Bashkir Curly
JTH: Japanese Thoroughbreds
