# Correction: Review: Genomic insights into the adaptive traits and stress resistance in modern horses

**DOI:** 10.1007/s44154-026-00327-z

**Published:** 2026-07-20

**Authors:** Halima Jafari, Belete Kuraz Abebe, Li Cong, Zulfiqar Ahmed, Wang Zhaofei, Minhao Sun, Gemingguli Muhatai, Lei Chuzhao, Ruihua Dang

**Affiliations:** 1 Key Laboratory of Animal Genetics, Breeding and Reproduction of Shaanxi Province, College of Animal Science and Technology, Northwest A&F University, Yangling, 712100 China; 2 Faculty of Veterinary and Animal Sciences, University of Poonch Rawalakot, Rawalakot, Azad Jammu and Kashmir 12350 Pakistan; 3 College of Animal Science and Technology, Tarim University, Alar, 843300 China

**Correction: Stress Biol 6, 5 (2026)**

**https://doi.org/10.1007/s44154-025-00274-1**

Following publication of the original article (Jafari et al. 2026), Fig. 1 has been updated to meet standard graphic representation requirements.

The original article (Jafari et al. 2026) has been updated.

## References

[CR1] Jafari H, Abebe BK, Cong L et al (2026) Review: Genomic insights into the adaptive traits and stress resistance in modern horses. Stress Biol 6:5. 10.1007/s44154-025-00274-1 41521281 10.1007/s44154-025-00274-1 PMC12791104

